# Peer Mentoring Improves Diabetes Technology Use and Reduces Diabetes Distress Among Underserved Communities: Outcomes of a Pilot Diabetes Support Coach Intervention

**DOI:** 10.1155/jdr/1970247

**Published:** 2025-07-09

**Authors:** Jennifer Maizel, Michael J. Haller, David M. Maahs, Ananta Addala, Stephanie L. Filipp, Rayhan A. Lal, Matthew J. Gurka, Lauren Figg, Melanie Hechavarria, Dessi P. Zaharieva, Keilecia G. Malden, Sarah Westen, Brittney N. Dixon, Korey Hood, Eleni Sheehan, Jessie J. Wong, William T. Donahoo, Marina Basina, Angelina Bernier, Eliana Frank, Ashby F. Walker

**Affiliations:** ^1^Department of Health Services Research, Management, and Policy, University of Florida College of Public Health and Health Professions, Gainesville, Florida, USA; ^2^Department of Medicine, University of Miami Miller School of Medicine, Miami, Florida, USA; ^3^Department of Pediatrics, Division of Endocrinology, University of Florida College of Medicine, Gainesville, Florida, USA; ^4^Division of Pediatric Endocrinology and Diabetes, Stanford University School of Medicine, Stanford, California, USA; ^5^Division of Endocrinology, Gerontology, and Metabolism, Stanford University School of Medicine, Stanford, California, USA; ^6^Department of Public Health Sciences, University of Virginia School of Medicine, Charlottesville, Virginia, USA; ^7^Department of Clinical and Health Psychology, University of Florida College of Public Health and Health Professions, Gainesville, Florida, USA; ^8^Division of Psychiatry and Behavioral Sciences, Stanford University School of Medicine, Stanford, California, USA; ^9^Department of Medicine, Division of Endocrinology, Diabetes, and Metabolism, University of Florida College of Medicine, Gainesville, Florida, USA

**Keywords:** diabetes, health coaching, health equity, peer support, social support

## Abstract

**Background:** There are well-documented disparities in diabetes care outcomes and technology usage, stemming from differences in healthcare access, distrust in healthcare providers, and other factors. This study evaluated patient-level outcomes of a diabetes support coach (DSC) intervention aimed at improving underserved adults' diabetes technology use, diabetes distress, and HbA1c levels.

**Methods:** As part of a Project Extension for Community Healthcare Outcomes (ECHO) Diabetes program, a social support intervention involving 28 DSCs was piloted at 33 Federally Qualified Health Centers (FQHCs) in Florida and California from May 2021 to May 2022. DSCs, who were adults with diabetes, served in a capacity similar to peer mentors and community health workers and received uniform training/oversight by a clinical team. Intervention participants (*n* = 74 adults with insulin-requiring diabetes at FQHCs) self-enrolled and engaged with DSCs via text messages, phone calls, and events. Participants' outcomes were evaluated cross-sectionally via the Diabetes Distress Scale (DDS-17) and a diabetes technology usage survey and longitudinally via HbA1c tests upon enrollment and at 6-month follow-up. A group of adults with insulin-requiring diabetes from the same FQHCs who did not receive the DSC intervention (*n* = 363) was used for comparison. Descriptive statistics were computed for all outcomes (*n*, percentage; mean, SD/95% CI). Between-group comparisons were evaluated via chi-squared and *t*-tests.

**Results:** DSC intervention participants reported significantly lower diabetes distress than the comparison group (DDS-17 score mean = 1.6 vs. 2.1, *p* < 0.001), and significantly more participants in the DSC intervention regularly used continuous glucose monitors (CGMs) than the comparison group (69.9% vs. 38.8%, *p* < 0.0001). There were no significant differences in insulin pump usage or HbA1c.

**Conclusions:** Lower diabetes distress and greater CGM usage among intervention participants suggest that the DSCs' shared lived experiences and healthcare navigation support positively influenced underserved adults' outcomes. These findings show DSCs' potential for improving diabetes care and technology equity.

## 1. Introduction

Access to social support is crucial for people with diabetes, as these individuals face unique health-related challenges that are often associated with higher stress, distress, and isolation [1, 2]. Social support involves the provision of assistance or aid exchanged through interpersonal relationships and can be emotional, instrumental, informational, or appraisal-focused [1, 3]. Among people with diabetes, social support can serve as a motivational pathway for improving diabetes self-management attitudes, behaviors, and glycemic outcomes [1].

Early studies examining the role of social support in diabetes self-management found that patients who reported higher social support had lower hemoglobin A1c (HbA1c) levels and better adherence to medication and dietary regimens [4, 5]. More recent studies have elucidated the value of peer support and mentoring in diabetes self-management [6–10]. Peer support and mentoring are social support methods that involve transferring experiential knowledge of a specific behavior or coping strategy for a stressor between people who share a characteristic, such as having type 1 diabetes (T1D) or type 2 diabetes (T2D) [11]. Interventions and resources that promote peer support enable people with diabetes to communicate with others who have the same condition; thus, they feel less alone and more understood [6]. Additionally, recent research has examined the role of community health workers (CHWs) in providing social support to people with diabetes [12, 13]. CHWs are professionals employed in settings such as community health centers and public health departments, and they have a close connection with the communities they serve [12]. CHWs provide culturally competent health education, outreach, and other forms of social support to individuals affected by a variety of health issues in their area [14]. CHWs can complete or provide diabetes-specific programs; however, they are not necessarily licensed or credentialed in diabetes care [12].

Research indicates that people with diabetes who received peer support or CHW support reported lower HbA1c levels, improved diabetes self-management skills and confidence, and better health-related quality of life [9, 10, 13]. Although peer mentors and CHWs both have been shown to improve outcomes for people with diabetes, they offer distinct forms of social support. Peer mentors provide shared personal experiences living with diabetes while CHWs typically provide healthcare navigation and informational services [12].

Underserved communities, including racial and ethnic minorities, under- and uninsured individuals, and individuals residing in geographically underresourced areas, have less access to social support [15–17]. Underserved communities with diabetes also have higher HbA1cs, diabetes distress, and mortality rates and lower utilization of new therapeutic approaches and technologies including insulin pumps and continuous glucose monitoring (CGM) systems, which can improve glycemic outcomes [18–21]. Furthermore, underserved communities with diabetes have lower access to endocrinologists due to geographic and socioeconomic barriers; thus, they are more likely to see primary care providers who often lack diabetes-specific training and resources to provide optimal diabetes care support [22, 23]. Underserved populations also report greater distrust in healthcare and are more receptive to medical guidance provided by representatives and peers from their own communities [12]. As such, there is an urgent need for social support interventions targeting underserved communities with diabetes, especially recently in the context of the COVID-19 pandemic, when these individuals reported heightened isolation and other psychosocial issues [24–26].

Project Extension for Community Healthcare Outcomes (ECHO) Diabetes is a tele-education and social support intervention that is aimed at improving outcomes for underserved adults with diabetes receiving care at Federally Qualified Health Centers (FQHCs) and similar community primary care centers. It is one of over 30 diabetes-related programs in the United States that use the Project ECHO model [27–31]. Project ECHO Diabetes enrolled 33 health centers throughout Florida and California, which were strategically recruited through usage of the National Deprivation Index (NDI) and geocoding efforts focused in areas with high poverty and few endocrinologists [32, 33]. Project ECHO Diabetes employed the standard Project ECHO “hub-and-spoke” model, through which multidisciplinary core “hub” teams of clinical experts in Florida and California, including endocrinologists, diabetes psychologists, dietitians, exercise physiologists, pharmacists, and other specialists, provided specialty care resources to the “spoke” sites, which are the FQHCs and community primary care centers [27, 34]. Participating spokes engaged in videoconferences featuring didactic lectures and case presentations on a weekly basis for the first 6 months and then bimonthly after 6 months of enrollment. The spokes received other resources, including online educational materials and access to diabetes support coaches (DSCs), adults living with T1D and T2D who acted as peer mentors and CHW-like healthcare navigators for underserved patients [27].

The DSCs provided support to underserved patients regarding their adoption and usage of new diabetes technologies and therapeutics as well as their lifestyle behaviors and psychosocial well-being. The DSCs lived local to the FQHCs and were able to provide guidance regarding state and region-specific diabetes care resources; several also shared participants' racial and ethnic backgrounds. The DSCs completed specialized training in health coaching via the University of California San Francisco's Center for Excellence in Primary Care and the Diabetes Paraprofessional Level 1 certification from the American Association of Diabetes Care and Education Specialists. They also received professional membership resources designed for CHWs from the American Diabetes Association. The DSCs' integration of peer mentors' lived experiences and CHWs' healthcare navigation skills presents a comprehensive approach to social support in diabetes care. Furthermore, utilization of DSCs in the Project ECHO Diabetes program is novel, given that CHWs and peer mentors are not core tenants of the Project ECHO model and this has the potential to strengthen its patient-level impacts.

**Figure 1:**
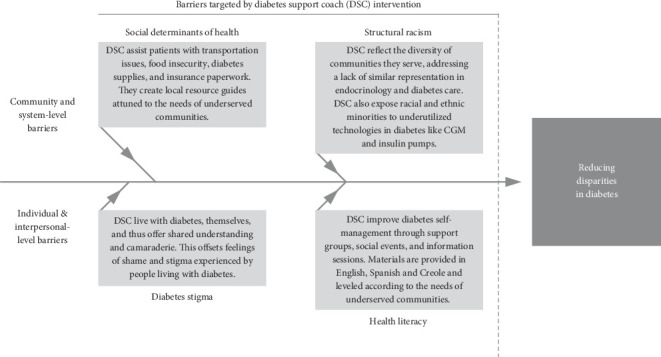
Reducing health disparities in diabetes through diabetes support coaching. From Walker et al. Using Peer Power to Reduce Health Disparities: Implementation of a Diabetes Support Coach Program in Federally Qualified Health Centers. Diabetes Spectrum 2022; 35(3); doi:10.2337/dsi22-0004. Copyright 2022, American Diabetes Association.

Usage of the DSC intervention in the Project ECHO Diabetes program has been published elsewhere along with a conceptual model (Figure 1) illustrating how this program targets key social barriers for underserved communities [35]. In preliminary research, spoke site primary care providers reported on program exit surveys that the DSCs were effective at sustaining their patients' engagement in diabetes self-management [27]. However, research assessing patient-level outcomes is needed to evaluate the effectiveness of the DSC intervention.

To fill this gap, the current study is a patient-level outcomes' evaluation of the DSC intervention, piloted from May 2021 to May 2022. Specific outcomes measured in this study included patients' diabetes distress, diabetes technology utilization, and HbA1c levels. Through this evaluation, we analyzed the effectiveness of DSCs in improving CGM and insulin pump utilization and overall therapeutic outcomes among underserved adults with T1D and insulin-requiring T2D.

## 2. Materials and Methods

### 2.1. Procedure

As part of an overall evaluation of the Project ECHO Diabetes program, adults aged ≥ 18 years with T1D and T2D on multiple daily injections (MDIs) of insulin were recruited for survey research at participating spoke sites through a process of informed consent and protocols overseen by the Institutional Review Boards (IRBs) of each hub team [32]. All study procedures were approved by the University of Florida and Stanford University IRBs.

Participating spoke sites provided patient lists for recruitment based on the following eligibility criteria: (1) diagnosis of T1D or T2D, (2) using intensive insulin management, (3) ages ≥ 18 years, and (4) seen for care at the spoke site twice in the past year or once in each of the past two consecutive years. The patient lists were randomized upon receipt to ensure no bias in the order in which patients were contacted. The lists were then used for recruitment via phone or at the spoke sites by dedicated study staff that had first-hand knowledge of working with underserved communities and were bilingual in English and Spanish [32]. Baseline demographic characteristics were obtained from the participants and the spoke sites' electronic health record (EHR) upon enrollment using Research Electronic Data Capture (REDCap). The Single-Item Literacy Screener (SILS) was also used [36].

Participants completed surveys at baseline and 6 or 12 months postenrollment using REDCap survey links or, depending on preference, pencil/paper versions that were mailed with return postage-provided envelopes. Surveys included a range of topics, including questions on social support systems, diabetes technology use, diabetes care, diabetes-related complications, and the Diabetes Distress Scale (DDS-17) [37]. Participants received $20 compensation for each survey they completed. HbA1c home testing kits were also mailed at 6-month intervals postenrollment. HbA1c data were also extracted from the health centers' EHR systems for patients who were unable to complete a home testing kit during the evaluation period [38]. The Advanced Research and Diagnostic Laboratory at the University of Minnesota provided and processed the HbA1c kits [39].

Participants recruited for this study included those who opted to work with a DSC and those who did not work with a DSC. All participating spoke sites had access to a DSC, and patients' entry into the DSC intervention was through self-enrollment at social events hosted at the spokes, in the community, or virtually, or based on recommendation to a patient by a participating provider. Each DSC provided social support to their assigned mentees through regular contact via phone outreach (text messages and calls, expected to occur weekly) and at in-person clinic visits or in-person or virtual social events. The DSCs provided mentees with guidance regarding medication management, stress-coping strategies, diet regimens, and physical activity as well as assisted with scheduling medical appointments, disseminated information about CGM systems and insulin pumps, and developed diabetes resource guides. The DSCs did not offer direct medical advice. All DSC encounters were documented in REDCap, including the date/time, length of the visit, modality of interaction (text vs. in-person, etc.), the main concerns of the mentees, and how the issue was addressed.

### 2.2. Analysis

Data analysis for this study compared DDS-17 outcomes, survey items pertaining to technology usage, and HbA1c levels between participants working with a DSC and other patients with insulin-requiring diabetes at the spokes who were not. Data pertaining to demographics and DSC encounters were also analyzed. All data analysis was computed in SAS (SAS Institute). *p* values < 0.05 were considered statistically significant.

Demographic characteristics and DSC encounters were analyzed using descriptive statistics (frequencies, percentages, means, and standard deviations (SDs)). Between-group demographic comparisons were made using the Cochran–Mantel–Haenszel (CMH) general association test for all variables except for age, a continuous variable in which a *t*-test was used, and sex assigned at birth, a binary variable for which a chi-squared test was used. For the DDS-17, frequencies and percentages were tabulated for all individual item responses, and the Cochran–Armitage test for trend assessed for between-group differences in frequencies of item scores. Total DDS-17 scores were calculated only for participants who responded to all 17 items, and total scores for the four DDS-17 subscales [37] were calculated only for participants who responded to all respective items. Chi-squared tests determined between-group differences in frequencies of participants who scored above the clinical threshold for diabetes distress (≥ 3 = moderate diabetes distress) [37]. *t*-tests analyzed for between-group differences in participants' score on the full DDS-17 and four subscales (mean score divided by the number of questions); the Sattherwaite method was used for the physician-related distress subscale due to unequal variances. Chi-squared tests assessed for between-group differences in diabetes technology usage. Due to limited survey responses and staggered participant enrollment into the DSC intervention, DDS-17 and diabetes technology usage data from both time points were combined and evaluated cross-sectionally. Between-group comparisons of HbA1c data were analyzed using *t*-tests. HbA1c data were also compared within groups by race and ethnicity (White, Black, Hispanic, or other) using a *t*-test with the Satterthwaite method due to unequal variances and by state (Florida or California) using an ANOVA. HbA1c data were evaluated longitudinally at baseline and 6-month follow-up.

## 3. Results

The total sample included 437 participants; 16.9% (*n* = 74) were in the DSC intervention group and 83.1% (*n* = 363) were in the comparison group. The number of individual responses to each question on the DDS-17 and diabetes technology usage survey varied since all questions were optional. Of the 437 participants, 259 had HbA1c data available for comparison (intervention group *n* = 58; comparison group *n* = 201).

### 3.1. Demographics

Between-group differences in demographic characteristics were not statistically significant; as such, demographic data for all participants are presented (Table 1). The mean age of all participants was 52.6 years + /−SD 14.2 years (*n* = 430). Regarding type of diabetes, 57.9% (*n* = 250) had T2D, 37.7% (*n* = 163) had T1D, 3.5% (*n* = 15) had latent autoimmune diabetes in adults (LADA), and 0.9% (*n* = 4) had other types. Participants were 54.5% (*n* = 237) female and 45.5% (*n* = 198) male. Participants were 48.3% (*n* = 208) Non-Hispanic White, 21.4% (*n* = 92) Non-Hispanic Black, 20.9% (*n* = 90) Hispanic, 4.9% (*n* = 21) other/multiracial, 2.6% (*n* = 11) American Indian/Alaska Native (AI/AN), 1.6% (*n* = 7) Asian, and 0.5% (*n* = 2) Native Hawaiian/Pacific Islander (NHPI). Participants' educational attainment varied: 29.4% (*n* = 126) received a high school diploma or GED, 22% (*n* = 94) completed some college but did not receive a degree, 14.7% (*n* = 63) received a bachelor's degree, 13.6% (*n* = 58) completed some high school education but did not receive a diploma, 11.5% (*n* = 49) completed an associate's degree, 4.9% (*n* = 21) completed a master's degree, 0.7% (*n* = 3) received a professional degree, and 3.3% (*n* = 14) did not specify their educational attainment. Most participants used Medicare (30.8%, *n* = 130), Medicaid (25.8%, *n* = 109), or commercial insurance (23.2%, *n* = 98), while others were uninsured and self-paid (12.3%, *n* = 52), used dual eligible Medicare/Medicaid (5.9%, *n* = 25), or used Indian Health Service (IHS) insurance (1.9%, *n* = 8).

### 3.2. DSC Encounters

During the study period, a total of 2006 communications between DSCs and intervention participants occurred. Most communications were via phone calls (*n* = 748, 37.3%), text messages (*n* = 600, 29.9%), at indoor meetings at nonmedical public places (*n* = 237, 11.8%), and at clinic visits (*n* = 150, 7.5%). Most encounters were between 15 and 30 min (*n* = 594, 29.6%), < 15 min (*n* = 508, 25.3%), between 46 and 60 min (*n* = 303, 15.1%), and between 31 and 45 min (*n* = 284, 14.2%). The DSC encounters primarily involved following up on an established action plan (*n* = 786, 39.2%), discussing participants' questions or concerns (*n* = 618, 30.8%), agenda setting (*n* = 266, 13.3%), and discussing participants' diabetes stories (*n* = 256, 12.8%). Most participants' questions and concerns pertained to medications (*n* = 525, 26.2%), food (*n* = 512, 25.5%), stress (*n* = 338, 16.9%), and exercise (*n* = 333, 16.6%). DSC encounter data are displayed in Table 2. A thematic analysis of participants' open-ended feedback pertaining to “other” types of questions and concerns (*n* = 230) will be completed in a subsequent study.

### 3.3. Diabetes Distress

DSC intervention participants (*n* = 70) had a significantly lower overall mean DDS-17 item score than the comparison group (*n* = 343; mean = 1.6, 95%CI = [1.1, 2.4] vs. mean = 2.1, 95%CI = [1.4, 3.1]; *p* = 0.0077). DSC intervention participants (*n* = 70) also had significantly lower scores on the emotional burden, physician-related distress, and regimen-related distress DDS-17 subscales than the comparison group (*n* = 343); for emotional burden, the mean item score among DSC intervention participants was 2.0 (95%CI = [1.2, 3.2]), while the mean item score for the comparison group was 2.6 (95%CI = [1.6, 4.2]; *p* = 0.043). For physician-related distress, intervention participants had a lower mean item score = 1 (95%CI = [1, 1.5]) than the comparison group (mean item score = 1; 95%CI = [1, 2]; *p* = 0.0129). For regimen-related distress, the mean item score for the intervention group was 1.8 (95%CI = [1.2, 2.4]), while the comparison group's mean item score was 2.4 (95%CI = [1.4, 3.6]; *p* = 0.0023). There was no significant difference for interpersonal distress; however, the intervention group had a lower mean item score of 1.0 (95%CI = [1, 2]) than the comparison group (1.3; 95%CI = [1, 2.7]). DDS-17 scores are presented in Table 3.

There were also significant between-group differences in four individual item score frequencies on the DDS-17. For the item, “feeling that I will end up with serious long-term complications, no matter what I do,” the DSC intervention group had a lower frequency of scores ≥ 4, which would indicate “somewhat serious” diabetes distress worthy of clinical attention (intervention group: 23.6%; *n* = 17 vs. comparison group: 40.9%; *n* = 147; *p* = 0.0106). For the item, “feeling that I am often failing with my diabetes management routine,” 20.6% (*n* = 15) of intervention participants scored ≥ 4, versus 34.4% (*n* = 124) in the comparison group (*p* = 0.0479). For the item, “feeling that I am not sticking closely enough to a good meal plan,” 28.7% (*n* = 21) of intervention participants scored ≥ 3, a lower percentage than in the control group (51.6%, *n* = 156, *p* < 0.0001). Lastly, for the item, “not feeling motivated to keep up my diabetes self-management,” 27.8% (*n* = 20) of intervention participants scored ≥ 3, versus 39.3% (*n* = 142) in the comparison group (*p* = 0.0123).

### 3.4. Diabetes Technology Usage

Participants in the DSC intervention reported significantly higher CGM utilization than the comparison group (69.9% vs. 38.8%, *n* = 51 vs. *n* = 140; *p* < 0.0001). There was no significant difference in insulin pump usage; however, more DSC intervention participants reported ever using these devices than those in the comparison group (28.8% vs. 22.7%; *n* = 21 vs. *n* = 82, respectively). Diabetes technology usage comparisons are reported in Table 3.

### 3.5. HbA1c Levels

Participants in the DSC intervention (*n* = 58) had a mean baseline HbA1c of 8.3% (95% CI = [7.3%, 10.2%]) and a mean 6-month follow-up HbA1c of 8.0% (95%CI = [7.2%, 9.8%]), with a mean change of −0.1% (95%CI = [−0.8%, 0.3%]). Participants in the comparison group (*n* = 201) had a mean baseline HbA1c of 7.6% (95%CI = [6.9%, 8.9%]) and a mean 6-month follow-up HbA1c also of 7.6% (95%CI = [6.7%, 8.9%]), with a mean change of −0.1% (95%CI = [−0.5%, 0.5%]). The between-group difference in mean HbA1c change was not statistically significant. There were also no significant racial and ethnic or state differences in HbA1c levels within the two groups. HbA1c data is presented in Table 3.

## 4. Discussion

This study evaluated patient-level outcomes of a pilot peer support intervention using DSCs for underserved adults with insulin-requiring diabetes. Overarching results demonstrated that DSCs were valuable for reducing diabetes distress and improving use of CGM systems among underserved communities over a 6-month intervention period. Specifically, participants in the DSC intervention had lower mean DDS-17 item scores and fewer instances of “somewhat serious,” “serious,” or “very serious” diabetes distress pertaining to fear of long-term complications, feelings of failure associated with diabetes management, concerns about adherence to meal plans, and motivation to keep up with diabetes self-management. Regarding CGM system utilization, the percentage of participants in the DSC intervention who used these devices at least once per month was nearly double that of the comparison group. Although between-group HbA1c differences were not statistically significant, it is important to note that the difference in mean baseline HbA1c was clinically meaningful. Specifically, DSC intervention participants had a 0.7 higher mean HbA1c than those in the comparison group. This suggests that higher HbA1c levels may have influenced participants' decision to enroll in the intervention, as they may have felt DSCs would help them with their diabetes management.

These findings demonstrate that social support provided by the DSCs improved participants' perceptions of their own diabetes self-management skills and self-efficacy. As adults living with T1D and T2D themselves, the DSCs were able to provide empathetic guidance to underserved adults, as well as share insights regarding their own diabetes management challenges and strategies. These types of insights would not typically be shared by healthcare providers. Most of the DSCs also wore CGM devices and were able to share insights regarding their own experiences with CGM as well as connect their mentees with sensor samples, when available. As underserved communities report greater distrust in healthcare providers, the DSCs' perspectives as peer mentors with lived experiences likely contributed to participants' willingness to utilize these devices. Similarly, the DSCs' ability to connect participants to CGM sensor samples and other diabetes care resources helped to reduce accessibility barriers faced by underserved adults. Furthermore, as the DSCs lived in the same geographic areas as intervention participants and several DSCs shared the intervention participants' racial and ethnic backgrounds, they were able to serve as local healthcare navigators and trusted community messengers. These findings are supported by earlier qualitative research with the DSCs [35].

Further research is needed to evaluate the longer-term impacts of the DSC intervention on participants as well as the experiences of the DSCs themselves. A larger study of the entire Project ECHO Diabetes' stepped wedge trial demonstrated a statistically significant increase in both CGM use and insulin pump use among adults with T1D and T2D using MDI at all participating FQHCs [40]. Beyond this, longer-term analyses are needed to determine if changes in CGM usage among program participants in this current study are sustained with or without ongoing exposure to DSCs. Given this study's promising results, it would also be valuable for future research to examine the DSCs' impact on uptake of automated insulin delivery systems among this population. Qualitative analysis is also needed to yield deeper insights into this study's findings as well as intervention participants' experiences in the DSC program. A preliminary qualitative study found that the DSCs had positive experiences in this program [35]; however, follow-up research is needed to evaluate the DSCs' longer-term experiences and perspectives.

To our knowledge, this study is the first patient-level outcomes evaluation of a DSC intervention designed for underserved communities with T1D and T2D at FQHCs and community primary care centers. This multisite intervention provided patients at these health centers with all four types of social support; emotional and appraisal support was offered through regular interactions between people living with diabetes; informational support was given through healthcare resource guides and lifestyle tips; and instrumental support was facilitated through assistance with scheduling medical appointments and locating relevant diabetes care resources [3]. The DSC intervention also provided online social support to high-risk patients during the COVID-19 pandemic, a time when they reported higher distress and increased social isolation [24].

Regarding this study's limitations, all data except for HbA1c levels were self-reported. Participants' responses on the DDS-17 and diabetes technology survey could have been biased or made in error due to factors such as social desirability and inaccurate recall [41]. All survey questions were optional; as such, some participants did not provide a response to each question. Therefore, outcomes identified in this evaluation may be exaggerated or underreported. Given the small sample of intervention participants, their staggered enrollment into the intervention, and short duration of the pilot study, survey and DDS-17 data were limited and therefore had to be evaluated cross-sectionally. Additionally, while there is a larger program evaluation of Project ECHO Diabetes involving randomization of new health centers in a stepped-wedge design [32, 40], this particular study of the DSC intervention did not employ randomization at the patient level for enrollment. As such, results may be influenced by self-selection bias. Although not statistically significant, there is a possibility that confounding factors between the intervention and comparison groups, such as demographic differences, affected the results we observed. This study also lacked qualitative methods, which would have yielded deeper insights into patients' experiences in the DSC program and health outcomes. However, as noted previously, ongoing evaluations of the Project ECHO Diabetes and DSC programs have incorporated qualitative data collection, and their findings will be published in subsequent studies.

## 5. Conclusions

This study serves as an important first step in understanding patient-level outcomes of the pilot DSC intervention. Results demonstrate that peer mentoring and CHW support are valuable for underserved communities with diabetes and underscore the broader success of the Project ECHO Diabetes intervention [40]. The DSC intervention significantly reduced diabetes distress and increased CGM system usage among underserved adults with diabetes, who historically have reported higher distress and barriers to CGM system access. The DSC intervention's overall success also underscores the importance of online social support for people with diabetes during the COVID-19 pandemic, when they experienced more social isolation. Further research utilizing longitudinal and qualitative methods is needed to assess the long-term impacts of the DSC intervention and the lived experiences of participants.

## Figures and Tables

**Table 1:** Participant demographic characteristics.

	**All (** **N** = 437**)**		**No DSC (** **N** = 363**)**		**DSC (** **N** = 74**)**		**p** ** value**
	**N**	**n** ** (%), ** **m** **e** **a** **n** ± **STD**	**N**	**n** ** (%), ** **m** **e** **a** **n** ± **STD**	**N**	**n** ** (%), ** **m** **e** **a** **n** ± **STD**	
Age	430	52.6 ± 14.2	359	52.6 ± 14.3	71	52.9 ± 13.7	0.8487
*Sex at birth*	435		362		73		0.1370
Male		198 (45.5)		159 (43.9)		39 (53.4)	
Female		237 (54.5)		203 (56.1)		34 (46.6)	
*Diabetes type*	432		360		72		0.8129
Type 1 diabetes		163 (37.7)		135 (37.5)		28 (38.9)	
Type 2 diabetes		250 (57.9)		208 (57.8)		42 (58.3)	
Latent autoimmune diabetes		15 (3.5)		13 (3.6)		2 (2.8)	
Other		4 (0.9)		4 (1.1)		—	
*Insurance coverage*	422		351		71		0.1480
Commercial		98 (23.2)		86 (24.5)		12 (16.9)	
Medicare		130 (30.8)		101 (28.8)		29 (40.9)	
Medicaid		109 (25.8)		89 (25.4)		20 (28.2)	
Dual eligible Medicare/Medicaid		25 (5.9)		20 (5.7)		5 (7.0)	
Indian Health Service		8 (1.9)		8 (2.3)		—	
Uninsured/self-pay		52 (12.3)		47 (13.4)		5 (7.0)	
*Race/ethnicity*	431		357		74		0.0577
White		208 (48.3)		180 (50.4)		28 (37.8)	
Black		92 (21.4)		67 (18.8)		25 (33.8)	
Hispanic		90 (20.9)		72 (20.2)		18 (24.3)	
Asian		7 (1.6)		6 (1.7)		1 (1.4)	
Native HI/Pacific Islander		2 (0.5)		2 (0.6)		—	
American Indian/Alaska Native		11 (2.6)		10 (2.8)		1 (1.4)	
Other/multiple		21 (4.9)		20 (5.6)		1 (1.4)	
*Education*	428		358		70		0.5093
Some high school, no diploma		58 (13.6)		51 (14.3)		7 (10.0)	
High school diploma or GED		126 (29.4)		98 (27.4)		28 (40.0)	
Some college, no degree		94 (22.0)		80 (22.4)		14 (20.0)	
Associate degree		49 (11.5)		43 (12.0)		6 (8.6)	
Bachelor's degree		63 (14.7)		53 (14.8)		10 (14.3)	
Master's degree		21 (4.9)		17 (4.8)		4 (5.7)	
Professional degree		3 (0.7)		3 (0.8)		—	
Do not know or do not wish to provide		14 (3.3)		13 (3.6)		1 (1.4)	

**Table 2:** DSC encounter summaries.

**Encounters**	**n** ** (%)**
*Communications*	2006
Clinic visit	150 (7.5)
Individual, nonmedical visit: Patient home	64 (3.2)
Individual, nonmedical visit: Public, indoor	237 (11.8)
Individual, nonmedical visit: Public, outdoor	65 (3.2)
Phone call	748 (37.3)
Zoom video call	21 (1.1)
Email	66 (3.3)
ECHO social gathering	9 (0.5)
Text	600 (29.9)
Not detailed	46 (2.3)
*Communication time*	
< 15 min	508 (25.3)
15–30 min	594 (29.6)
31–45 min	284 (14.2)
46–60 min	303 (15.1)
60+ min	246 (12.3)
Not detailed	71 (3.5)
*Tasks conducted^a^*	
Agenda setting	266 (13.3)
Follow-up on action plan	786 (39.2)
Create new action plan	174 (8.7)
Discuss concerns or questions	618 (30.8)
Discuss patient's diabetes stories	423 (21.1)
Discuss coach's diabetes stories	256 (12.8)
Upcoming visit reminder	138 (6.9)
Assist with paperwork	72 (3.6)
Alert triage nurse or provider	30 (1.5)
Notify of diabetes social gathering	133 (6.6)
Other	234 (11.7)
*Patient expressed concerns or had questions regarding^a^*	
Medications	525 (26.2)
Food	512 (25.5)
Exercise	333 (16.6)
Stress	338 (16.9)
A1c	144 (7.2)
Blood pressure	28 (1.4)
Cholesterol	27 (1.4)
Weight	86 (4.3)
Working with the provider	190 (9.5)
Using the clinic/resources	147 (7.3)
Other	230 (11.5)

^a^Check all that apply, individual rates presented in rows for each.

**Table 3:** Participants' technology usage, HbA1c levels, and DDS-17 scores.

	**All**	**No DSC**	**DSC**	**p** ** value**
*Technology use*	*N* = 434	*N* = 361	*N* = 73	
Have you ever used an insulin pump? *n* (%)				
Yes	103 (23.7)	82 (22.7)	21 (28.8)	0.2676
No	331 (76.3)	279 (77.3)	52 (71.2)	
Do you regularly (at least once a month) use a CGM? *n* (%)				
Yes	191 (44.0)	140 (38.8)	51 (69.9)	< 0.0001⁣^∗^
No	243 (56.0)	221 (61.2)	22 (30.1)	
*HbA1c*	*N* = 259	*N* = 201	*N* = 58	
Baseline Month 0 measurement mean [95% CI]	7.7 [6.9, 9.2]	7.6 [6.9, 8.9]	8.3 [7.3, 10.2]	
Follow-up Month 6 measurement mean [95% CI]	7.7 [6.8, 9.1]	7.6 [6.7, 8.9]	8.0 [7.2, 9.8]	
HbA1c 6-month change mean [95% CI]	−0.1 [−0.5, 0.5]	−0.1 [−0.5, 0.5]	−0.1 [−0.8, 0.3]	0.2527
*Diabetes Distress Scale*	*N* = 413	*N* = 343	*N* = 70	
Total DDS: Item score mean [95% CI]	2.0 [1.4, 3.1]	2.1 [1.4, 3.1]	1.6 [1.1, 2.4]	0.0077⁣^∗^
*n* (%) above range (≥ 3)	111 (26.9)	97 (28.3)	14 (20.0)	0.1544
*DDS-17 subscales*	*N* = 413	*N* = 343	*N* = 70	
Emotional burden				
Item score mean [95% CI]	2.4 [1.6, 4.0]	2.6 [1.6, 4.2]	2.0 [1.2, 3.2]	0.0430⁣^∗^
*n* (%) above range (≥ 3)	163 (39.5)	140 (40.8)	23 (32.9)	0.2144
Physician-related distress				
Item score mean [95% CI]	1.0 [1.0, 2.0]	1.0 [1.0, 2.0]	1.0 [1.0, 1.5]	0.0129⁣^∗^
*n* (%) above range (≥ 3)	58 (14.0)	52 (15.2)	6 (8.6)	0.1482
Regimen-related distress				
Item score mean [95% CI]	2.2 [1.4, 3.4]	2.4 [1.4, 3.6]	1.8 [1.2, 2.4]	0.0023⁣^∗^
*n* (%) above range (≥ 3)	141 (34.1)	126 (36.7)	15 (21.4)	0.0138⁣^∗^
Interpersonal distress				
Item score mean [95% CI]	1.3 [1.0, 2.3]	1.3 [1.0, 2.7]	1.0 [1.0, 2.0]	0.1300
*n* (%) above range (≥ 3)	92 (22.3)	80 (23.3)	12 (17.1)	0.2574

⁣^∗^ denotes statistical significance.

## Data Availability

Data are available upon reasonable request by directly reaching out to the ECHO Diabetes PIs: Dr. Ashby Walker (afwalker@ufl.edu), Dr. David Maahs (dmaahs@stanford.edu), or Dr. Michael Haller (hallemj@peds.ufl.edu).

## References

[1] Rad G. S., Bakht L. A., Feizi A., Mohebi S. (2013). Importance of Social Support in Diabetes Care. *Journal of Education and Health Promotion*.

[2] Ducat L., Philipson L. H., Anderson B. J. (2014). The Mental Health Comorbidities of Diabetes. *JAMA*.

[3] Holt-Lunstad J., Uchino B. N., Glanz B. K. R., Viswanath K. V. (2015). Social Support and Health. *Health Behavior: Theory, Research, and Practice*.

[4] Garay-Sevilla M. E., Nava L. E., Malacara J. M. (1995). Adherence to Treatment and Social Support in Patients With Non-Insulin Dependent Diabetes Mellitus. *Journal of Diabetes and its Complications*.

[5] Ilias I., Hatzimichelakis E., Souvatzoglou A., Anagnostopoulou T., Tselebis A. (2001). Perception of Family Support Is Correlated With Glycemic Control in Greeks With Diabetes Mellitus. *Psychol Rep*.

[6] Heisler M. (2007). Overview of Peer Support Models to Improve Diabetes Self-Management and Clinical Outcomes. *Diabetes Spectrum*.

[7] Yin J., Wong R., Au S. (2015). Effects of Providing Peer Support on Diabetes Management in People With Type 2 Diabetes. *Annals of Family Medicine*.

[8] Walker A. F., Haller M. J., Gurka M. J. (2020). Addressing Health Disparities in Type 1 Diabetes Through Peer Mentorship. *Pediatr Diabetes*.

[9] Litchman M. L., Edelman L. S., Donaldson G. W. (2018). Effect of Diabetes Online Community Engagement on Health Indicators: Cross-Sectional Study. *JMIR Diabetes*.

[10] Litchman M. L., Allen N. A., Sanchez-Birkhead A. (2022). Continuous Glucose Monitoring Plus an Online Peer Support Community Reinforces Healthy Behaviors in Hispanic Adults With Type 2 Diabetes. *Diabetes Spectrum*.

[11] Boothroyd R. I., Fisher E. B. (2010). Peers for Progress: Promoting Peer Support for Health Around the World. *Family Practice*.

[12] Walker A. F., Graham S., Maple-Brown L. (2023). Interventions to Address Global Inequity in Diabetes: International Progress. *Lancet*.

[13] Palmas W., March D., Darakjy S. (2015). Community Health Worker Interventions to Improve Glycemic Control in People With Diabetes: A Systematic Review and Meta-Analysis. *Journal of General Internal Medicine*.

[14] Landers S. J., Stover G. N. (2011). Community Health Workers—Practice and Promise. *Am J Public Health*.

[15] Walker A. F., Schatz D. A., Johnson C., Silverstein J. H., Rohrs H. J. (2015). Disparities in Social Support Systems for Youths With Type 1 Diabetes. *Clinical Diabetes*.

[16] Gauthier G. R., Smith J. A., García C., Garcia M. A., Thomas P. A. (2021). Exacerbating Inequalities: Social Networks, Racial/Ethnic Disparities, and the COVID-19 Pandemic in the United States. *Journals of Gerontology: Series B*.

[17] Kim G., Dautovich N., Ford K.-L. (2017). Geographic Variation in Mental Health Care Disparities Among Racially/Ethnically Diverse Adults With Psychiatric Disorders. *Soc Psychiatry Psychiatr Epidemiol*.

[18] Agarwal S., Kanapka L. G., Raymond J. K. (2020). Racial-Ethnic Inequity in Young Adults With Type 1 Diabetes. *The Journal of Clinical Endocrinology & Metabolism*.

[19] Saydah S., Imperatore G., Cheng Y., Geiss L. S., Albright A. (2017). Disparities in Diabetes Deaths Among Children and Adolescents — United States, 2000–2014. *MMWR Morb Mortal Wkly Rep*.

[20] Helgeson V. S., Naqvi J. B., Korytkowski M. T., Gary-Webb T. L. (2021). A Closer Look at Racial Differences in Diabetes Outcomes Among a Community Sample: Diabetes Distress, Self-Care, and HbA1c. *Diabetes Care*.

[21] Kanbour S., Jones M., Abusamaan M. S. (2023). Racial Disparities in Access and Use of Diabetes Technology Among Adult Patients With Type 1 Diabetes in a U.S. Academic Medical Center. *Diabetes Care*.

[22] Romeo G. R., Hirsch I. B., Lash R. W., Gabbay R. A. (2020). Trends in the Endocrinology Fellowship Recruitment: Reasons for Concern and Possible Interventions. *The Journal of Clinical Endocrinology & Metabolism*.

[23] Walker A. F., Hood K. K., Gurka M. J. (2021). Barriers to Technology Use and Endocrinology Care for Underserved Communities With Type 1 Diabetes. *Diabetes Care*.

[24] Maizel J. L., Dixon B. N., Walker A. F. (2023). Psychological Outcomes of the COVID-19 Pandemic on People With Type 1 Diabetes Globally: A Scoping Review. *Current Diabetes Reviews*.

[25] Cyranka K., Dudek D., Małecki M. T., Matejko B., Klupa T. (2021). Psychological Crisis Intervention for COVID-19 Lockdown Stress in Patients With Type 1 Diabetes Mellitus: Survey Study and Qualitative Analysis. *JMIR Ment Health*.

[26] Maizel J. L., Haller M. J., Maahs D. M. (2024). COVID-19 Impacts and Inequities Among Underserved Communities With Diabetes. *Journal of Clinical & Translational Endocrinology*.

[27] Walker A. F., Cuttriss N., Haller M. J. (2021). Democratizing Type 1 Diabetes Specialty Care in the Primary Care Setting to Reduce Health Disparities: Project Extension for Community Healthcare Outcomes (ECHO) T1D. *BMJ Open Diabetes Research & Care*.

[28] Health Resources and Services Administration Health Center Program Award Recipients|HRSA. https://www.hrsa.gov/opa/eligibility-and-registration/health-centers/fqhc.

[29] Arora S., Thornton K., Murata G. (2011). Outcomes of Treatment for Hepatitis C Virus Infection by Primary Care Providers. *N Engl J Med*.

[30] Arora S., Thornton K., Komaromy M., Kalishman S., Katzman J., Duhigg D. (2014). Demonopolizing Medical Knowledge. *Academic Medicine*.

[31] Cloutier B., Trevino D. (2022). Project ECHO Annual Report 2022. https://digitalrepository.unm.edu/hsc_echo_ar/1.

[32] Addala A., Hechavarria M., Figg L. (2023). Recruiting Historically Under-Represented Individuals Into Project ECHO Diabetes: Using Barrier Analysis to Understand Disparities in Clinical Research in the USA. *BMJ Open*.

[33] Messer L. C., Laraia B. A., Kaufman J. S. (2006). The Development of a Standardized Neighborhood Deprivation Index. *Journal of Urban Health*.

[34] Arora S., Geppert C. M. A., Kalishman S. (2007). Academic Health Center Management of Chronic Diseases Through Knowledge Networks: Project ECHO. *Academic Medicine*.

[35] Walker A. F., Addala A., Sheehan E. (2022). Using Peer Power to Reduce Health Disparities: Implementation of a Diabetes Support Coach Program in Federally Qualified Health Centers. *Diabetes Spectrum*.

[36] Morris N. S., MacLean C. D., Chew L. D., Littenberg B. (2006). The Single Item Literacy Screener: Evaluation of a Brief Instrument to Identify Limited Reading Ability. *BMC Family Practice*.

[37] Polonsky W. H., Fisher L., Earles J. (2005). Assessing Psychosocial Distress in Diabetes. *Diabetes Care*.

[38] Little R. R., Rohlfing C. L., Sacks D. B., National Glycohemoglobin Standardization Program (NGSP) Steering Committee (2011). Status of Hemoglobin A1c Measurement and Goals for Improvement: From Chaos to Order for Improving Diabetes Care. *Clinical Chemistry*.

[39] The Diabetes Control and Complications Research Group (1987). Diabetes Control and Complications Trial (DCCT): Results of Feasibility Study. The DCCT Research Group. *Diabetes Care*.

[40] Walker A. F., Haller M. J., Addala A. (2025). Project ECHO Diabetes Trial Improves Outcomes for Medically Underserved People. *Diabetes Care*.

[41] Althubaiti A. (2016). Information Bias in Health Research: Definition, Pitfalls, and Adjustment Methods. *Journal of Multidisciplinary Healthcare*.

